# Combine and conquer: manganese synergizing anti-TGF-β/PD-L1 bispecific antibody YM101 to overcome immunotherapy resistance in non-inflamed cancers

**DOI:** 10.1186/s13045-021-01155-6

**Published:** 2021-09-15

**Authors:** Ming Yi, Mengke Niu, Jing Zhang, Shiyu Li, Shuangli Zhu, Yongxiang Yan, Ning Li, Pengfei Zhou, Qian Chu, Kongming Wu

**Affiliations:** 1 Department of Oncology, Tongji Hospital of Tongji Medical College, Huazhong University of Science and Technology, 1095 Jiefang Avenue, Wuhan, 430030 People’s Republic of China; 2 Wuhan YZY Biopharma Co., Ltd, Biolake, C2-1, No.666 Gaoxin Road, Wuhan, 430075 People’s Republic of China; 3 Department of Medical Oncology, The Affiliated Cancer Hospital of Zhengzhou University & Henan Cancer Hospital, Zhengzhou, 450008 People’s Republic of China

**Keywords:** The tumor microenvironment, Manganese, cGAS-STING, PD-L1, TGF-β, Bispecific antibody, Cancer immunotherapy

## Abstract

**Background:**

Our previous work showed that the anti-TGF-β/PD-L1 bispecific antibody YM101 effectively overcame anti-PD-L1 resistance in immune-excluded tumor models. However, in immune-desert models, the efficacy of YM101 was limited. Bivalent manganese (Mn^2+^) is identified as a natural stimulator of interferon genes (STING) agonist, which might enhance cancer antigen presentation and improve the therapeutic effect of YM101.

**Methods:**

The effect of Mn^2+^ on STING pathway was validated by western blotting and enzyme-linked immunosorbent assay. Dendritic cell (DC) maturation was measured by flow cytometry. The synergistic effect between Mn^2+^ and YM101 in vitro was determined by one-way mixed lymphocyte reaction, CFSE dilution assay, and cytokine detection. The in vivo antitumor effect of Mn^2+^ plus YM101 therapy was assessed in CT26, EMT-6, H22, and B16 tumor models. Flow cytometry, RNA-seq, and immunofluorescent staining were adopted to investigate the alterations in the tumor microenvironment.

**Results:**

Mn^2+^ could activate STING pathway and promote the maturation of human and murine DC. The results of one-way mixed lymphocyte reaction showed that Mn^2+^ synergized YM101 in T cell activation. Moreover, in multiple syngeneic murine tumor models, Mn^2+^ plus YM101 therapy exhibited a durable antitumor effect and prolonged the survival of tumor-bearing mice. Relative to YM101 monotherapy and Mn^2+^ plus anti-PD-L1 therapy, Mn^2+^ plus YM101 treatment had a more powerful antitumor effect and a broader antitumor spectrum. Mechanistically, Mn^2+^ plus YM101 strategy simultaneously regulated multiple components in the antitumor immunity and drove the shift from immune-excluded or immune-desert to immune-inflamed tumors. The investigation in the TME indicated Mn^2+^ plus YM101 strategy activated innate and adaptive immunity, enhanced cancer antigen presentation, and upregulated the density and function of tumor-infiltrating lymphocytes. This normalized TME and reinvigorated antitumor immunity contributed to the superior antitumor effect of the combination therapy.

**Conclusion:**

Combining Mn^2+^ with YM101 has a synergistic antitumor effect, effectively controlling tumor growth and prolonging the survival of tumor-bearing mice. This novel cocktail strategy has the potential to be a universal regimen for inflamed and non-inflamed tumors.

**Supplementary Information:**

The online version contains supplementary material available at 10.1186/s13045-021-01155-6.

## Background

For a robust antitumor immune response, a sequence of stepwise events should be appropriately initiated, proceeded, and propagated [1]. This cyclic process starts with antigen release and ends with tumor-killing by immune cells [2, 3]. Ideally, the immune system could recognize all malignantly transformed cells and inhibit tumorigenesis [4]. However, in some situations, the antitumor immune response is hampered by negative regulators [5–8]. As a result, malignantly transformed cells escape from immune clearance and develop into overt tumors [9].

Programmed cell death protein 1/programmed cell death ligand 1 (PD-1/PD-L1) is a vital immune checkpoint signal in maintaining immune homeostasis [10]. In the tumor microenvironment (TME), the PD-1/PD-L1 axis is commonly hijacked by cancer cells to restrain the activities of tumor-infiltrating lymphocytes (TILs) [11, 12]. Blocking the PD-1/PD-L1 axis restores T cells from exhausted status and reinitiates a self-sustaining antitumor immune response [13]. At present, anti-PD-1/PD-L1 antibodies (α-PD-1/PD-L1 Abs) have been successfully used to treat multiple types of cancers [14–21]. Although α-PD-1/PD-L1 strategies mobilize a durable and potent antitumor immunity in some cancer patients, most patients could not benefit from this therapy [22, 23]. For these patients, PD-1/PD-L1 is not the primary speed-limiting factor for antitumor immunity, and it is insufficient to normalize the dysregulated antitumor immune response by blocking the PD-1/PD-L1 axis [24].

TGF-β has a substantial influence on the immune system: inhibiting the proliferation and functions of T cells, inducing the differentiation of naïve T cells toward regulatory T cells, undermining the antigen presentation capability of dendritic cells (DCs), enhancing the functions of cancer-associated fibroblasts (CAFs), and impairing the activities of natural killer cells (NKs) [25]. Hyperactive TGF-β signal shapes an immunosuppressive microenvironment and contributes to α-PD-1/PD-L1 resistance [26]. Given the synergistic effect between TGF-β and PD-1/PD-L1 in promoting immune evasion, we developed the anti-TGF-β/PD-L1 bispecific antibody (α-TGF-β/PD-L1 BsAb) YM101 [27]. Our previous study showed that the antitumor effect of YM101 was significantly superior to that of α-PD-L1 in some high TGF-β tumor models [27]. However, like α-PD-1/PD-L1, YM101 had modest antitumor effects in poorly immunogenic tumor models.

The weakly immunogenic tumors are regarded as cold tumors or immune-desert tumors [5]. In this context, inadequate cancer antigen presentation and rare cancer-specific CD8^+^ T cells in the TME lead to the primary resistance to α-PD-1/PD-L1 [16]. Enhancing the functions of antigen presentation cells (APCs) promotes the generation of cancer-specific CD8^+^ T cells, which might relieve the primary resistance to α-PD-1/PD-L1 [28]. Recently, some studies indicated that an innate immune signal, stimulator of interferon genes (STING), might be a valuable target for cancer immunotherapy [29]. As the downstream production of STING pathway, type I interferon (IFN-I) promotes the maturation and function of DCs and strengthens the cytotoxicity of effector cells [30]. It has been verified that STING agonist could synergize α-PD-1/PD-L1 in cancer immunotherapy [31]. Even in poorly immunogenic tumor models such as B16, STING agonist combined with α-PD-1/PD-L1 therapy significantly prolonged survival of mice, relative to corresponding monotherapies [32]. Bivalent manganese (Mn^2+^) is identified as a natural STING agonist [33–35]. In a phase I clinical study, Mn^2+^ plus α-PD-1 treatment had an enhanced antitumor effect and manageable safety profile [36].

Hereto, we supposed that the cocktail strategy containing Mn^2+^ and YM101 would have a broader antitumor spectrum and better therapeutic efficacy by simultaneously boosting innate and adaptive immunity and overcoming multiple immunosuppressive factors. In this work, we explored the synergistic effect between Mn^2+^ and YM101 in T cell activation assays in vitro. Besides, we investigated the antitumor effect of Mn^2+^ plus YM101 in murine tumor models with high TGF-β or low immunogenicity.

## Materials and methods

### Cell lines and therapeutic Abs

Murine cancer cell lines CT26 (colon cancer), EMT-6 (breast cancer), H22 (hepatocellular carcinoma), and B16 (melanoma) were cultured with RPMI-1640 containing 10% fetal bovine serum. The therapeutic Abs and corresponding isotype, including YM101, α-murine PD-L1, α-TGF-β, and human IgG (hIgG), were provided by Wuhan YZY Biopharma. YM101 is an α-TGF-β/PD-L1 BsAb developed based on the Check-BODY™ platform [27].

### Western blotting

Protein was extracted and separated as described [37, 38]. Human-Reactive STING Pathway Antibody Sampler Kit (38,866, CST), Mouse-Reactive STING Pathway Antibody Sampler Kit (16,029, CST), and α-GAPDH (5174, CST) were used in this assay. SuperSignal™ West Pico PLUS (34,577, Thermo Scientific) was used for signal detection.

### Induction of DC

Human peripheral blood mononuclear cells (PBMCs) were obtained from healthy donors. CD14^+^ PBMCs were isolated from PBMCs by EasySep™ Human Monocyte Isolation Kit (19,359, Stemcell). Human monocyte-derived DCs (MoDCs) were induced from CD14^+^ PBMCs by ImmunoCult™ Dendritic Cell Culture Kit (10,985, Stemcell) according to the recommendations of manufacturers. Briefly, 2.5 × 10^5^/well monocytes were seeded in 6-well plates and cultured with differentiation medium for three days. Later, the supernatant was removed after centrifugation and replaced with fresh differentiation medium. The plates were further cultured for 2 days, and then, 20 ng/ml Lipopolysaccharides (LPS) (L2630, Sigma-Aldrich) or 1 mM manganese chloride (244,589, Sigma-Aldrich) was added. After incubation for 1 day, cells were collected for further flow cytometry assays. α-CD80 (305,208, BioLegend), α-CD86 (374,206, BioLegend), α-HLA-DR (307,606, BioLegend), and α-CD11c (561,355, BD Biosciences) Abs were used to measure the maturation of DCs. Moreover, PD-L1 level was also detected in the flow cytometry assays using α-PD-L1 Ab (374,512, BioLegend).

Murine bone marrow-derived DCs (BMDCs) were induced as described [36]. The differentiation medium contained murine granulocyte–macrophage colony-stimulating factor (GM-CSF) (20 ng/ml, 315–03-50UG, PeproTech) and murine IL-4 (20 ng/ml, 214–14-50UG, PeproTech). Bone marrow cells were obtained from BALB/c mice and seeded in tissue-culture treated T-25cm^2^ flasks with differentiation medium. 2 days later, the supernatant was replaced with fresh differentiation medium, and the suspension cells were discarded. On day 5, the supernatant was removed after centrifugation and replaced with fresh differentiation medium. Then, the cells were treated with 20 ng/ml LPS or 0.5 mM Mn^2+^ for 1 day. Then, cells were harvested for flow cytometry assays. α-CD80 (560,016, BD Biosciences), α-CD86 (105,008, BioLegend), α-I-A/I-E (107,608, BioLegend), α-CD11c (553,801, BD Biosciences), and α-PD-L1 (124,312, BioLegend) Abs were used in the flow cytometry assays.

### Enzyme-linked immunosorbent assay (ELISA) and cell viability evaluation

Immature human MoDCs (1 × 10^6^/ml) and murine BMDCs (2 × 10^6^/ml) were treated with Mn^2+^ for 1 day. The supernatants were harvested for ELISA. Human and murine IFN-β levels were measured by Human IFN-beta Quantikine ELISA Kit (DIFNB0, R&D) and Mouse IFN-beta Quantikine ELISA Kit (MIFNB0, R&D). The ELISAs were performed following the recommendations of manufactures. The suspension and adherent cells were collected to measure cell viability by AO/PI double staining (CS2-0106-5ML, Nexcelom Bioscience).

### One-way mixed lymphocyte reaction (MLR)

In the MLR, the simulator cells were saline-treated or Mn^2+^-treated murine BMDCs from BALB/c mice. The responder cells were splenocytes from C57BL/6 mice. Before the MLR, the stimulator cells were treated by Mitomycin-C (40 μg/ml, M5353, Sigma-Aldrich) for 30 min, and the responder cells were labeled with carboxyfluorescein diacetate succinimidyl ester (CFSE) (5 μM, 65–0850-84, ThermoFisher). The ratio of responder to stimulator was 2:1. Then, TGF-β1 and treatment Abs were added to the mixed cells. The mixed cells were cultured with RPMI-1640 containing 10% fetal bovine serum for 96 h. Subsequently, the mixed cells and supernatants were harvested to measure cell proliferation and cytokine levels. The ratios of daughter CD4^+^ and CD8^+^ T cells were determined by CFSE dilution assays (α-CD4: 100,516, BioLegend; α-CD8α: 563,068, BD Biosciences). The cytokine levels were measured by LEGENDplex™ MU Th Cytokine Panel (741,044, BioLegend).

### Murine tumor models

We explored the antitumor effect of Mn^2+^ combined with YM101 in multiple murine tumor models, including CT26, EMT-6, H22, and B16. All mice were treated every other day for 12 days. For Mn^2+^ administration, mice received 5 mg/kg Mn^2+^ intranasally or intratumorally. For Ab treatment, mice were treated with equivalent mole hIgG (6.6 mg/kg), α-PD-L1 (6.6 mg/kg), or YM101 (9 mg/kg) by intraperitoneal injection. Tumor growth was monitored every 2 days, and tumor volume was calculated as following: volume = 0.5 × length × width^2^. Mice were killed when tumor volume was over 2500 mm^3^ or when the experiment ended.

#### Subcutaneous CT26 model

BALB/c mice were subcutaneously inoculated in the right groin with 1 × 10^6^ CT26 cells on day 0. Tumor-bearing mice were randomized into six groups: Isotype control, Mn^2+^, α-PD-L1, Mn^2+^ plus α-PD-L1, YM101, and Mn^2+^ plus YM101 (8 mice for each group). Treatment started on day 4 when tumor volumes reached 50 ~ 100 mm^3^.

#### Orthotopic EMT-6 model

BALB/c mice were inoculated in the right mammary fat pad with 5 × 10^5^ EMT-6 cells on day 0. Treatment was initiated on day 4 when tumor volumes reached 50 mm^3^. We then explored the effect of the combination treatment on the survival of EMT-6-bearing mice. BALB/c mice were challenged with 3 × 10^5^ EMT-6 cells on day 0. Therapy was initiated on day 2. The survival status of mice was monitored for 45 days.

Subsequently, we investigated the durable antitumor effect of the combination treatment. BALB/c mice were challenged with 3 × 10^5^ EMT-6 cells on day 0. Therapy was initiated on day 1. One week after the last treatment, the untreated or cured mice were rechallenged with 3 × 10^5^ EMT-6 cells.

#### Subcutaneous H22 model

BALB/c mice were subcutaneously inoculated in the right groin with 2 × 10^6^ H22 cells on day 0. Tumor-bearing mice were randomized into four groups: Isotype control, Mn^2+^, YM101, and Mn^2+^ plus YM101 (8 mice for each group). Treatment started on day 4 when tumor volumes reached 50 mm^3^.

#### Subcutaneous B16 model

Besides immune-excluded model, we also evaluated the treatment efficacy in immune-desert model. C57BL/6 mice were subcutaneously inoculated with 2 × 10^5^ B16 cells on day 0. Treatment was started on day 1. Then, we investigated whether the combination therapy could prolong the survival of tumor-bearing mice in the weakly immunogenic tumor model. C57BL/6 mice were subcutaneously inoculated with 2 × 10^5^ B16 cells on day 0. Treatment was started on day 1. The survival status of mice was monitored for 4 weeks.

#### Lung metastatic B16 model

We explored the antitumor effect of the combination therapy in the lung metastatic B16 model. C57BL/6 mice were intravenously injected with 2 × 10^5^ B16 cells on day 0. Treatment was started on day 1. 21 days after inoculation, the mice were euthanized, and the lung tissues were collected for H&E staining.

### Flow cytometry for tumor-infiltrating lymphocytes (TILs)

Murine tumor tissues were scissored and digested with Collagenase B (1 mg/ml, 11,088,807,001, Roche), DNase I (0.5 mg/ml, abs47047435, Absin), and Hyaluronidase (1 mg/ml, abs47014926, Absin) at 37℃ for 1 h. Then, the prepared suspensions were filtered through 40 μm strainers (CSS013040, JET BIOFIL). Before staining, the cells were dyed with Fixable Viability Stain 780 (565,388, BD Biosciences) and blocked with Ultra-LEAF™ Purified α-mouse CD16/32 (101,339, BioLegend). The detection Abs used in this assay were α-CD45 (560,510, BD Biosciences), α-CD3e (562,600, BD Biosciences), α-CD8α (563,068, BD Biosciences), α-CD49b (740,363, BD Biosciences), α-Ki67 (556,027, BD Biosciences), α-Granzyme-B (372,204, BioLegend), α-CD107a (564,349, BD Biosciences), α-CD69 (566,500, BD Biosciences), α-CD25 (553,075, BD Biosciences), α-CD44 (561,859, BD Biosciences), α-TNF-α (563,943, BD Biosciences), α-Perforin (11–9392-82, ThermoFisher), α-CD11c (566,504, BD Biosciences), and α-I-A/I-E (107,608, BioLegend). The auxiliary reagents used in this assay were FOXP3/Transcription Factor Staining Buffer Set (00–5523-00, ThermoFisher), Brilliant Stain Buffer (563,794, BD Biosciences), and GolgiPlug (555,029, BD Biosciences). Total cell count per 100 mg tumor tissue was calculated with Vi-Cell Auto (Beckman Coulter). Flow cytometry was performed with BD FACSCelesta. FACS data were analyzed using Flowjo v10.

### Immunofluorescent (IF) staining

Tissues were fixed with 10% formalin for 48 h. Then, fixed tissues were dehydrated, and embedded, sectioned, and transferred to slides. Tyramide signal amplification-based IF staining was conducted as described [39]. The Abs targeting CD3 (ab231775, Abcam), CD4 (ab183685, Abcam), CD8 (ab217344, Abcam), p-TBK1 (5483, CST), TGF-β1 (ab215715, Abcam), α-SMA (ab7817, Abcam), Vimentin (10,366–1-AP, Proteintech), Collagen-I (BS-10423R, Bioss) were used in this assay. Immunofluorescent images were previewed with Caseviewer software, and the regions of interest were circled by two pathologists.

The quantitative analysis of IF staining was performed with ImageJ software. The expression level of one specific protein was measured by the integral optical density of fluorescence signal. The depth of T cell infiltration was measured as described [27]. Briefly, the infiltration depth of one T cell was determined by the scaled distance of the T cell to the nearest tumor border. For each region of interest, the infiltration depth was calculated as the mean infiltration depth of all involved T cells.

### RNA-seq assay

After six times of treatments, four samples were randomly selected from each group for RNA-seq. The reference genome was Mus_musculus.GRCm38. The mRNA was extracted by Trizol and enriched by Oligo (dT)-labeled beads. The cDNA library construction, treatment with unique identifier adapter, and further deep sequencing were performed by Wuhan Seqhealth. The differentially expressed genes (DEGs) were screen out by R software (version: 4.0.0) with the edgeR package. The visualization of DEG profile was performed with pheatmap package. Gene Set Enrichment Analysis (GSEA) was performed using R with packages enrichplot and ClusterProfiler [40]. The antitumor immunity-related signatures were constructed and scored as previously reported (Additional File 1: Table S1) [27]. For the calculation of signature scores, a pseudocount of 0.001 RPKM was added to all genes. The scores of immune signatures were compared using the ROAST algorithm [41].

### Statistical analyses

Statistical calculations were performed with GraphPad Prism 8. For the data with Gaussian distribution and equal variance, Student’s *t* test was adopted to compare the difference between two groups. For the data with Gaussian distribution and heteroscedasticity, Welch’s correction was adopted. For the data not coincident with normal distribution, Mann–Whitney test was used to compare the difference between two groups. All tests were two-sided, and a *p* value below 0.05 was regarded statistically significant.

## Results

### Mn^2+^ specifically activated STING pathway

**Fig. 1:**
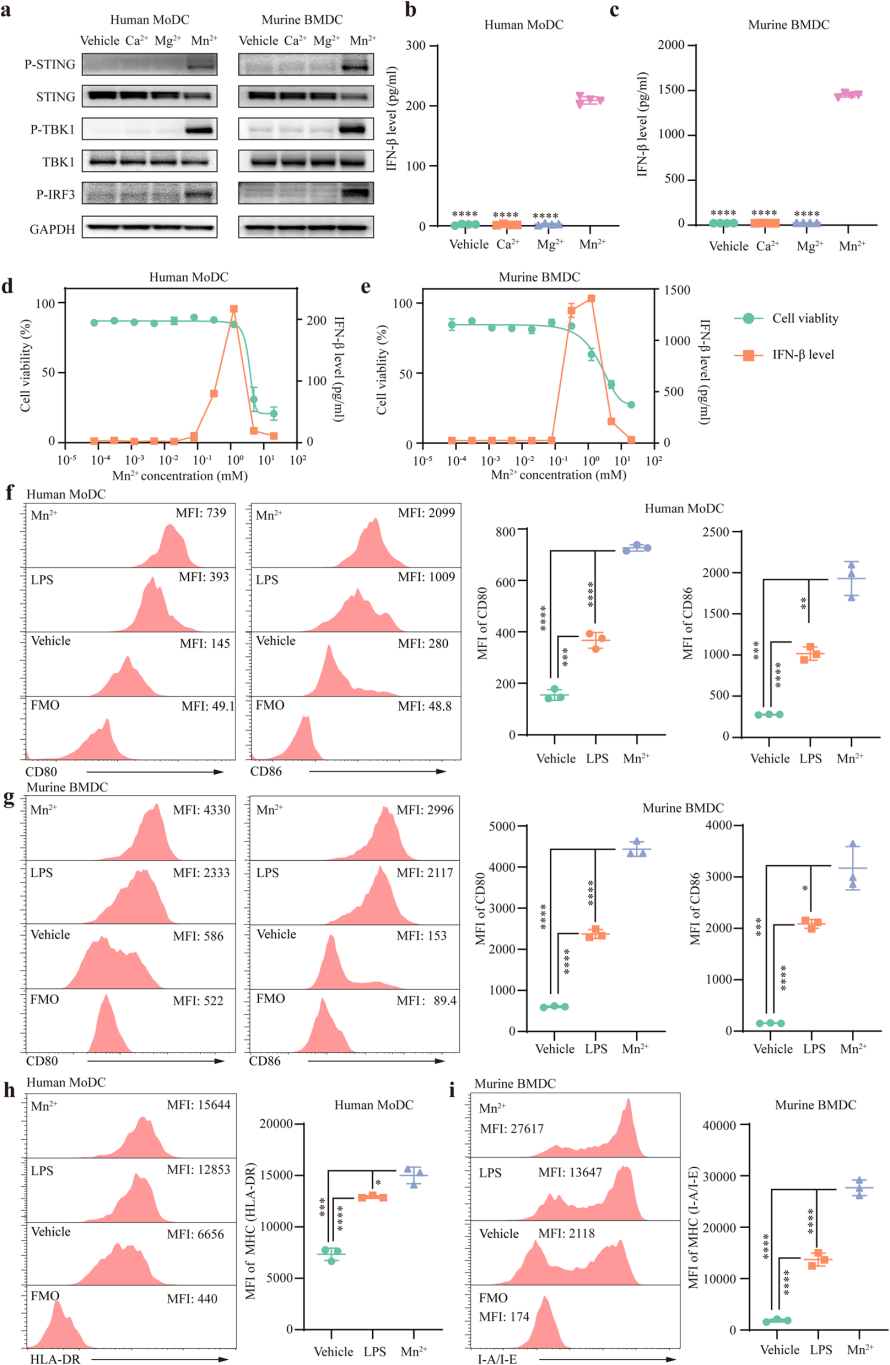
Mn^2+^ activated STING pathway and promoted dendritic cell (DC) maturation. **a**–**c** Western blotting assays and ELISAs exploring the effect of Mn^2+^ treatment on STING pathway in human monocyte-derived DCs (MoDCs) and murine bone marrow-derived DCs (BMDCs). After 1 mM Mn^2+^ or other divalent cations including Ca^2+^ and Mg^2+^ treatment for 24 h, the levels of p-STING, STING, p-TBK1, TBK1, p-IRF3, and IFN-β were measured. **d–e** The dose-dependent effect and toxicity of Mn^2+^ in vitro. Human MoDCs and murine BMDCs were treated with different concentrations of Mn^2+^ for 24 h. The IFN-β concentration in the supernatant was measured by ELISA, and the viability of MoDCs and BMDCs was determined by AO/PI double staining (n = 2 technical replicates). **f–i** Flow cytometry assays showed that Mn^2+^ promoted DC maturation. After 1 mM Mn^2+^ or 20 ng/ml LPS (positive control) treatment for 24 h, the mean fluorescence intensities (MFIs) of CD80, CD86, and HLA-DR on human MoDCs were measured. After 0.5 mM Mn^2+^ or 20 ng/ml LPS treatment for 24 h, the MFIs of CD80, CD86, and I-A/I-E on murine BMDCs were measured (n = 3 technical replicates). *p < 0.05, **p < 0.01, ***p < 0.001, and ****p < 0.0001 denote the significant difference relative to the last group when not marked with lines. MoDC: monocyte-derived dendritic cell; BMDC: bone marrow-derived dendritic cell; MFI: mean fluorescence intensity

We measured the effects of multiple divalent cations, including Ca^2+^, Mg^2+^, and Mn^2+^, on STING pathway. Among these divalent cations, only 1 mM Mn^2+^ activated STING pathway in MoDCs and BMDCs: promoting the phosphorylation of STING, TBK-1, and IRF-3, inducing the degradation of STING, and increasing the level of IFN-β (Fig. 1a-c). Then, we explored the dose-dependent effect and toxicity of Mn^2+^ in vitro. The gradient experiments showed that 1 mM or 0.5–1 mM Mn2+ led to an optimal activation of STING pathway in MoDCs or BMDCs without significant cytotoxicity (Fig. 1d and e). Therefore, in the following in vitro experiments, 1 mM or 0.5 mM Mn^2+^ was used to treat MoDCs or BMDCs.

### Mn^2+^ promoted the maturation of DC

Characterized by abundant co-stimulatory molecules and major histocompatibility complex (MHC), mature DCs had a stronger antigen presentation capability. To test the effect of Mn^2+^ on DC maturation, immature MoDCs and BMDCs were treated with Mn^2+^ or low-dose LPS for 24 h. We found that Mn^2+^ significantly increased the expression of co-stimulatory molecules CD80 and CD86 on DCs (Fig. 1f and g). Moreover, Mn^2+^ upregulated the level of major histocompatibility complex (MHC) on DCs: increasing HLA-DR on MoDCs and I-A/I-E on BMDCs (Fig. 1h and 1i). Our results indicated that Mn^2+^ effectively promoted the maturation of DCs.

### Mn^2+^ synergized YM101 in T cell activation

**Fig. 2:**
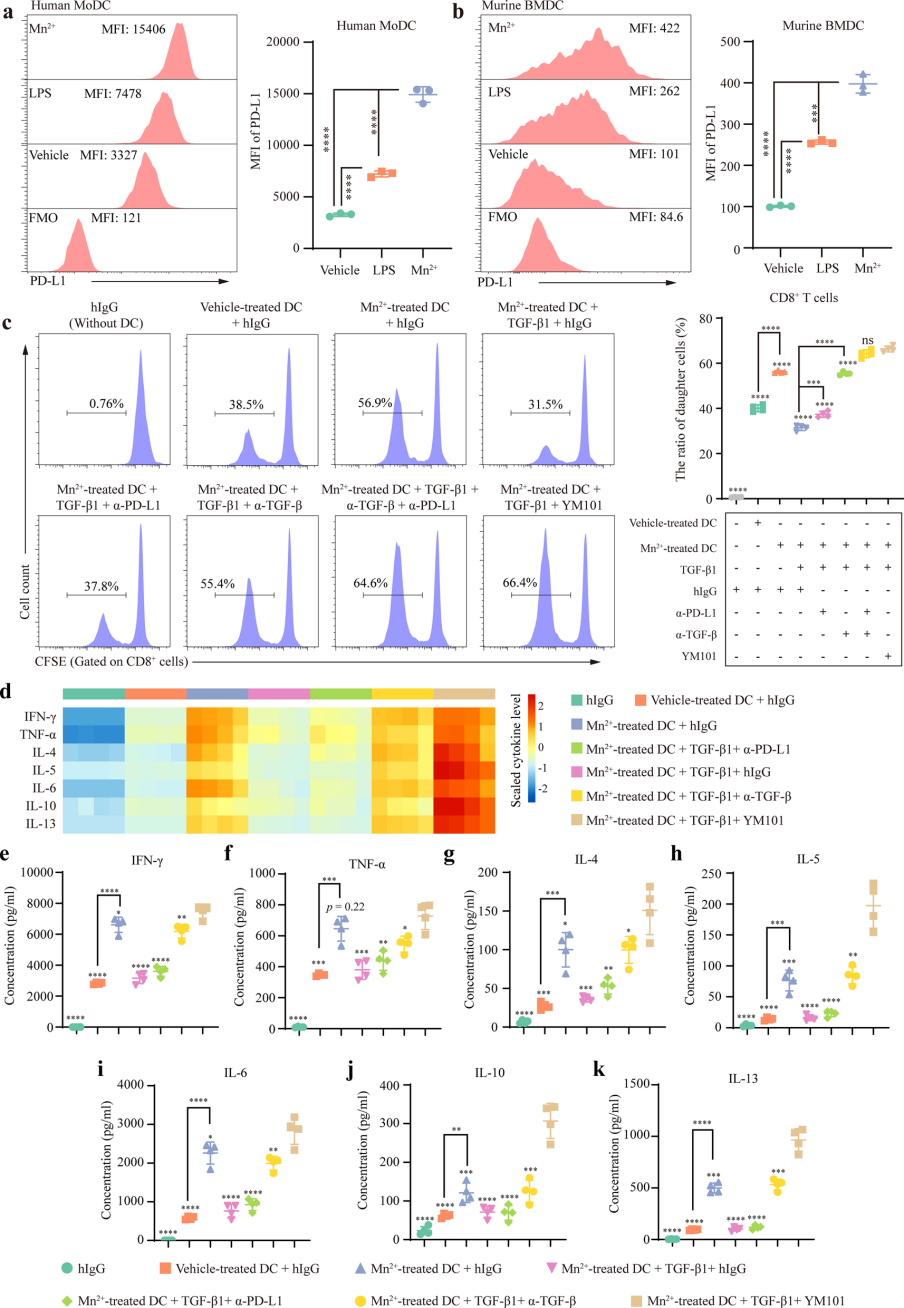
Mn^2+^ treatment upregulated PD-L1 and synergized YM101 in one-way mixed lymphocyte reaction (MLR) assay. **a–b** The effect of Mn^2+^ treatment on PD-L1 expression on dendritic cells (DCs). After 1 mM Mn^2+^ treatment for 24 h, the mean fluorescence intensity (MFI) of PD-L1 on MoDCs was measured. After 0.5 mM Mn^2+^ treatment for 24 h, MFI of PD-L1 on BMDCs was measured (n = 3 technical replicates). **c–k** MLR assay exploring the synergistic effect between Mn^2+^ treatment and YM101. BMDCs from BALB/c mice were used as stimulator cells, and splenocytes from C57BL/6 mice were used as responder cells. The immature BMDCs were treated with 0.5 mM Mn^2+^ or saline for 24 h. The ratio of responder to stimulator was 2:1. Then, TGF-β1 (20 ng/ml) and antibodies (10^5^ pM) were added to the mixed cells. After culture for 96 h, the ratio of daughter CD8^+^ T cell and the cytokine levels in the supernatant were measured (n = 4 technical replicates). The ratio of daughter CD8^+^ T cell by CFSE dilution assays (c) and the cytokine levels were measured by LEGENDplex™ MU Th Cytokine Panel. The cytokine levels were visualized by heatmap (d). The levels of IFN-γ, TNF-α, IL-4, IL-5, IL-6, IL-10, and IL-13 are shown (e–k). *p < 0.05, **p < 0.01, ***p < 0.001, and ****p < 0.0001 denote the significant difference relative to the last group when not marked with lines. MoDC: monocyte-derived dendritic cell; BMDC: bone marrow-derived dendritic cell; MFI: mean fluorescence intensity; MLR: mixed lymphocyte reaction

In parallel with increased co-stimulatory molecules and MHC, PD-L1 was also upregulated on DCs after Mn^2+^ treatment (Fig. 2a and b). Besides, our previous work had confirmed that TGF-β is an inhibitory factor for T cell activation [27]. Given this, we supposed that Mn^2+^ might synergize YM101 in a high TGF-β microenvironment by simultaneously enhancing antigen presentation and blocking immunosuppressive factors. Exogenous TGF-β1 was added to mimic a high TGF-β microenvironment, and one-way MLR was utilized to mimic T cell activation in the TME.

In this in vitro model, we found that Mn^2+^-treated DCs had a greater capability to present antigen and activate T cells. Relative to vehicle-treated DCs, Mn^2+^-treated DCs had a significant advantage in promoting CD4^+^ T (Additional File 1: Figure S1) and CD8^+^ T cell (Fig. 2c) proliferation. This advantage was weakened by exogenous TGF-β1. In the meanwhile, the TGF-β1-impaired T cell proliferation was partially restored by α-PD-L1 but almost entirely restored by α-TGF-β. Notably, α-PD-L1 plus α-TGF-β treatment and YM101 extended this advantage. In the MLR assay, CD8^+^ T cell had a higher proliferation potential than CD4^+^ T cell, consistent with previous reports [42].

Then, we investigated the cytokine panel during T cell activation to further confirm the synergistic effect between Mn^2+^ and YM101. Similarly, we found that Mn^2+^-treated DCs had a superior capability to stimulate cytokine secretion, relatively to saline-treated DCs (Fig. 2d). Besides, the DC-stimulated cytokine secretion was hampered by exogenous TGF-β1, and the TGF-β1-undermined cytokine secretion was reversed by α-TGF-β. Compared to α-TGF-β, YM101 further upregulated multiple Th1/Th2 cytokines, including IFN-γ, TNF-α, IL-4, IL-5, IL-6, IL-10, and IL-13 (Fig. 2e-2k).

### The potent antitumor effect of Mn^2+^ plus YM101

**Fig. 3:**
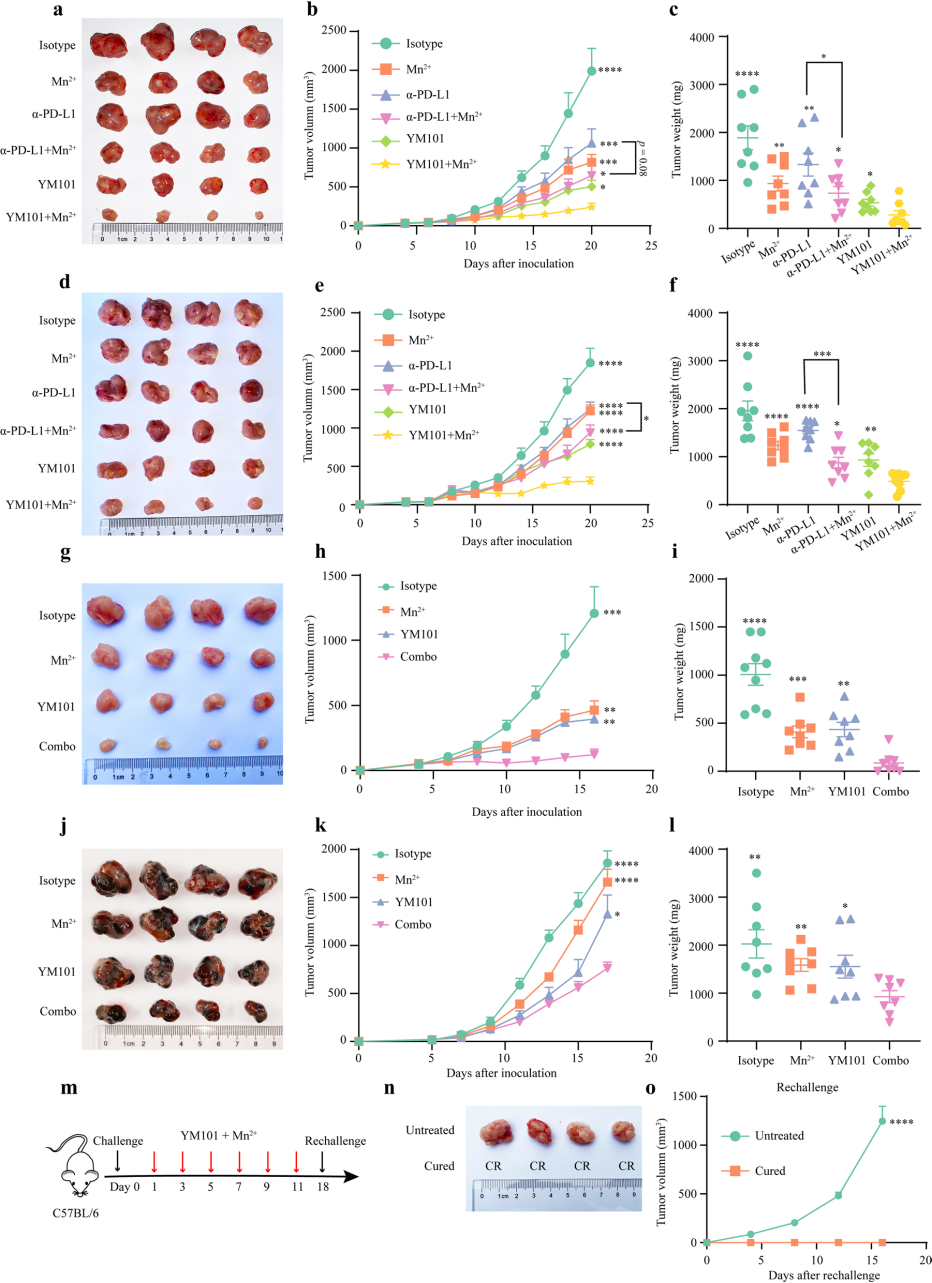
Mn^2+^ plus YM101 therapy had a potent and durable antitumor activity in vivo. All mice were treated every other day for 12 days. For Mn^2+^ administration, mice received 5 mg/kg Mn^2+^ intranasally or intratumorally. For Ab treatment, mice were treated with equivalent mole hIgG (6.6 mg/kg), α-PD-L1 (6.6 mg/kg), or YM101 (9 mg/kg) by intraperitoneal injection. **a–c** BALB/c mice were subcutaneously inoculated in the right groin with 1 × 10^6^ CT26 cells on day 0. Tumor-bearing mice were randomized into six groups: Isotype control, Mn^2+^, α-PD-L1, Mn^2+^ plus α-PD-L1, YM101, and Mn^2+^ plus YM101 (eight mice for each group). Treatment started on day 4. The representative images of CT26 tumors, tumor growth curves, and tumor weights are shown. **d–f** BALB/c mice were inoculated in the right mammary fat pad with 5 × 10^5^ EMT-6 cells on day 0. Tumor-bearing mice were randomized into six groups: Isotype control, Mn^2+^, α-PD-L1, Mn^2+^ plus α-PD-L1, YM101, and Mn^2+^ plus YM101 (eight mice for each group). Treatment was initiated on day 4. The representative images of EMT-6 tumors, tumor growth curves, and tumor weights are shown. **g–i** BALB/c mice were subcutaneously inoculated in the right groin with 2 × 10^6^ H22 cells on day 0. Tumor-bearing mice were randomized into four groups: Isotype control, Mn^2+^, YM101, and Mn^2+^ plus YM101 (eight mice for each group). Treatment started on day 4. The representative images of H22 tumors, tumor growth curves, and tumor weights are shown. **j–l** C57BL/6 mice were subcutaneously inoculated with 2 × 10^5^ B16 cells on day 0. Treatment was started on day 1 (eight mice for each group). The representative images of B16 tumors, tumor growth curves, and tumor weights are shown. **(m)** The schematic diagram of the rechallenge assay. BALB/c mice were inoculated with 3 × 10^5^ EMT-6 cells in the right mammary fat pad. Therapy was initiated on day 1. Mice received six times of treatments in 12 days. One week after the last treatment, the cured or treatment-naïve mice were rechallenged with 3 × 10^5^ EMT-6 cells. **(n–o)** The representative images of EMT-6 tumors and tumor growth curves in the rechallenge assay are shown. *p < 0.05, **p < 0.01, ***p < 0.001, and ****p < 0.0001 denote the significant difference relative to Mn^2+^ plus YM101 therapy when not marked with lines. CR: complete regression

A previous study showed that Mn^2+^ synergized α-PD-1/PD-L1 in multiple tumor models [36]. However, given the TGF-β-induced immunosuppression and treatment resistance, it was unclear if Mn^2+^ plus α-PD-L1 could effectively retard tumor growth in high TGF-β models. Theoretically, in this situation, Mn^2+^ combined with YM101 has a superior antitumor effect to Mn^2+^ plus α-PD-L1. It has been confirmed that CT26, EMT-6, and H22 are high TGF-β tumor models [43–45]. We assessed the antitumor effects of Mn^2+^, α-PD-L1, Mn^2+^ plus α-PD-L1, YM101, Mn^2+^ plus YM101 in the CT26, EMT-6, and H22 models. We found Mn^2+^ plus YM101 had the most potent antitumor activity among all treatment strategies: significantly inhibiting tumor growth and reducing tumor burden (Fig. 3a–3i). Our results indicated that the efficacy of Mn^2+^ plus YM101 was better than Mn^2+^ plus α-PD-L1 in high TGF-β tumor models. We then investigated the antitumor effect of Mn^2+^ plus YM101 in the B16 model, which is regarded as a weakly immunogenic tumor model. Although YM101 exhibited a limited antitumor effect in the B16 model, Mn^2+^ plus YM101 significantly suppressed tumor growth (Fig. 3j-3l).

Moreover, in the EMT-6 model, we tested the durability of antitumor effect. After six times of Mn^2+^ plus YM101 treatments, 4 out of 8 EMT-6-bearing mice were cured. A week later, the cured mice were rechallenged with EMT-6 cells. Mn^2+^ plus YM101 treatment showed a potent and sustaining antitumor activity, and the tumor growth was entirely inhibited in all cured mice (Fig. 3m-3o).

**Fig. 4:**
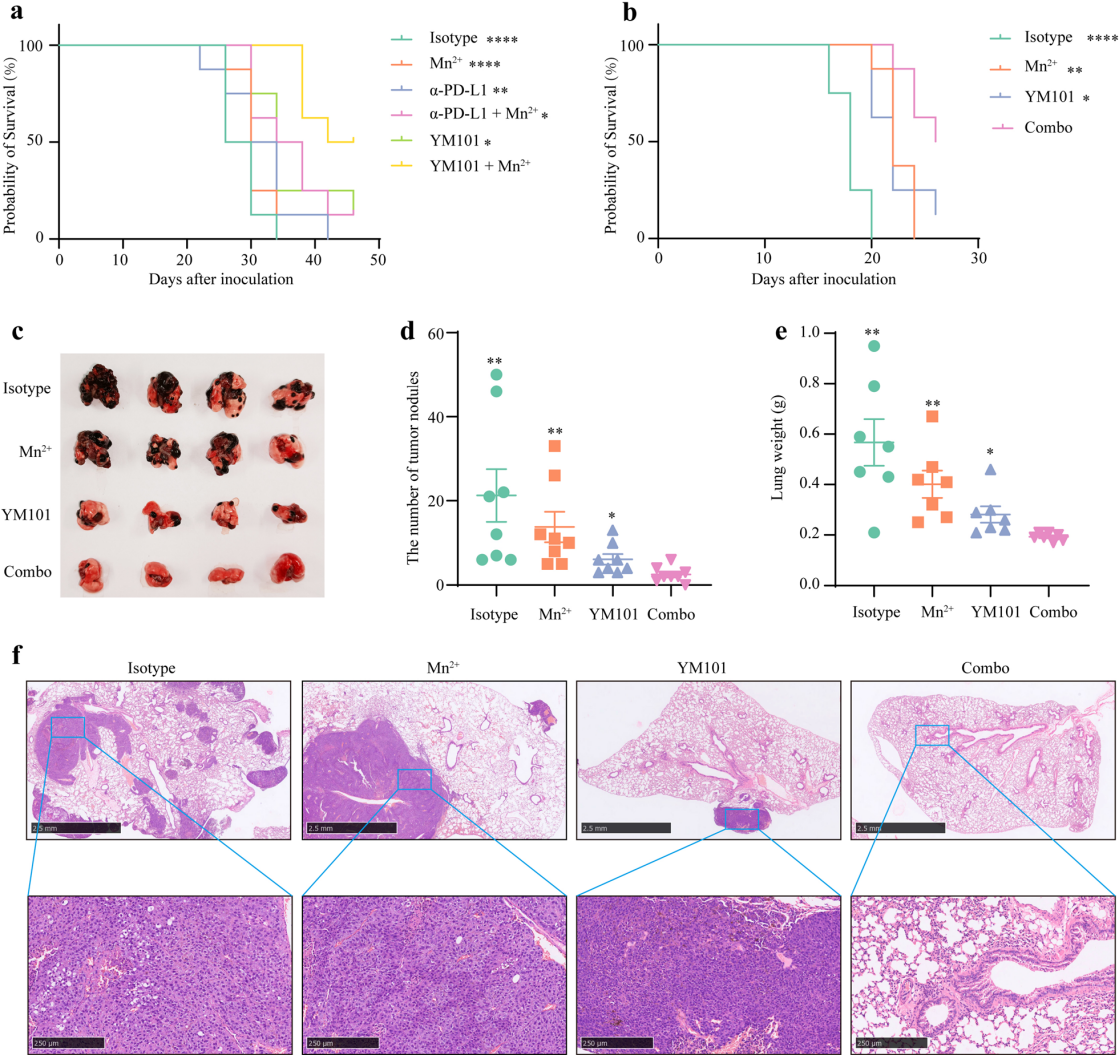
Mn^2+^ plus YM101 therapy prolonged survival and inhibited tumor metastasis. **a**–**b** The overall survival curves of EMT-6-bearing and B16-bearing mice receiving the treatment of combination therapies or monotherapies. BALB/c mice were challenged with 3 × 10^5^ EMT-6 cells on day 0. Therapy was initiated on day 2 (8 mice for each group). The survival status of mice was monitored for 45 days. C57BL/6 mice were subcutaneously inoculated with 2 × 10^5^ B16 cells on day 0. Treatment was started on day 1 (8 mice for each group). The survival status of mice was monitored for 4 weeks. **c** The representative images of lung tissues of mice receiving the treatment of combination therapies or monotherapies. C57BL/6 mice were intravenously injected with 2 × 10^5^ B16 cells on day 0. Treatment was started on day 1 (8 mice for each group). 21 days after inoculation, the mice were euthanized, and the lung tissues were collected. **d** The number of B16 tumor nodules in lung tissues of mice receiving the treatment of combination therapies or monotherapies. **e** The weights of lung tissues of mice receiving the treatment of combination therapies or monotherapies. **f** The representative images of H&E staining of lung tissues of mice receiving the treatment of combination therapies or monotherapies. *p < 0.05, **p < 0.01, ***p < 0.001, and ****p < 0.0001 denote the significant difference relative to combination treatment

Additionally, we explored the effect of Mn^2+^ plus YM101 treatment on the survival of tumor-bearing mice. In the EMT-6 and B16 models, Mn^2+^ plus YM101 dramatically prolonged survival (Fig. 4a and 4b). Besides subcutaneous and orthotopic models, we also tested the antitumor effect of Mn^2+^ plus YM101 therapy in the lung metastatic B16 model. 21 days after intravenous injection, murine lung tissues were harvested to measure the number of tumor nodules. We found YM101 partially reduced the number of metastatic foci, while Mn^2+^ plus YM101 treatment almost entirely inhibited the formation of metastasis (Fig. 4c-4f).

### Mn^2+^ plus YM101 treatment shaped an immunosupportive microenvironment

**Fig. 5:**
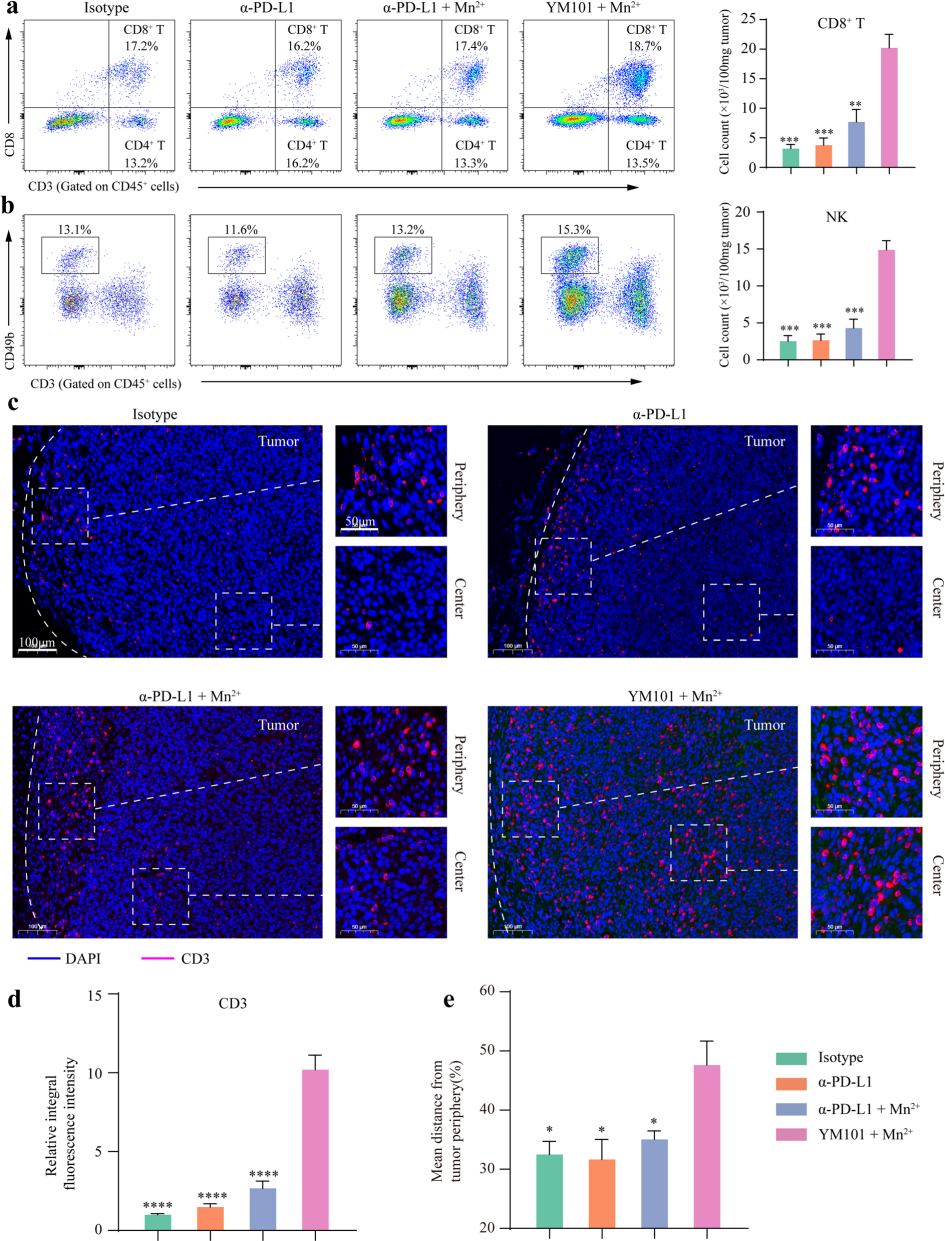
Mn^2+^ plus YM101 therapy promoted the transformation from immune-excluded to immune-inflamed tumors and enhanced immune cell infiltration in the EMT-6 model. **a** The representative flow cytometry images of tumor-infiltrating CD8^+^ T cells. **b** The representative flow cytometry images of tumor-infiltrating natural killer cells (NKs). **c** The representative images of α-CD3 staining in the tumor periphery and the tumor center. **d** The quantitative analysis for T cell number by integral fluorescence intensity. **e** The quantitative analysis for the depth of T cell infiltration (n = 5 biological replicates). *p < 0.05, **p < 0.01, ***p < 0.001, and ****p < 0.0001 denote the significant difference relative to Mn^2+^ plus YM101 therapy

In the EMT-6 model, relative to the Mn^2+^ plus α-PD-L1 group, the densities of tumor-infiltrating CD8^+^ and NK cells were significantly higher in the Mn^2+^ plus YM101 group (Fig. 5a and 5b). The results of IF indicated that compared to Mn^2+^ plus α-PD-L1 therapy, Mn^2+^ plus YM101 remarkedly decreased peritumoral collagen production (Additional File 1: Figure S2) and promoted T cell penetration (Fig. 5c). In the Mn^2+^ plus α-PD-L1 group, T cells were primarily distributed in tumor periphery. However, in the Mn^2+^ plus YM101 group, the location of T cells was not limited in tumor periphery, but also in tumor center. Mn^2+^ plus YM101 treatment increased the number of tumor-infiltrating T cells, as well as the depth of T cell infiltration (Fig. 5d and 5e). This finding was in accordance with our previous work [27]. Blocking TGF-β signaling led to the conversion from immune-excluded to immune-inflamed tumors, providing an optimal immune cell positioning for antitumor immune response. The enhanced immune cell infiltration by α-TGF-β moiety might contribute to better cancer control in the Mn^2+^ plus YM101 group.

**Fig. 6:**
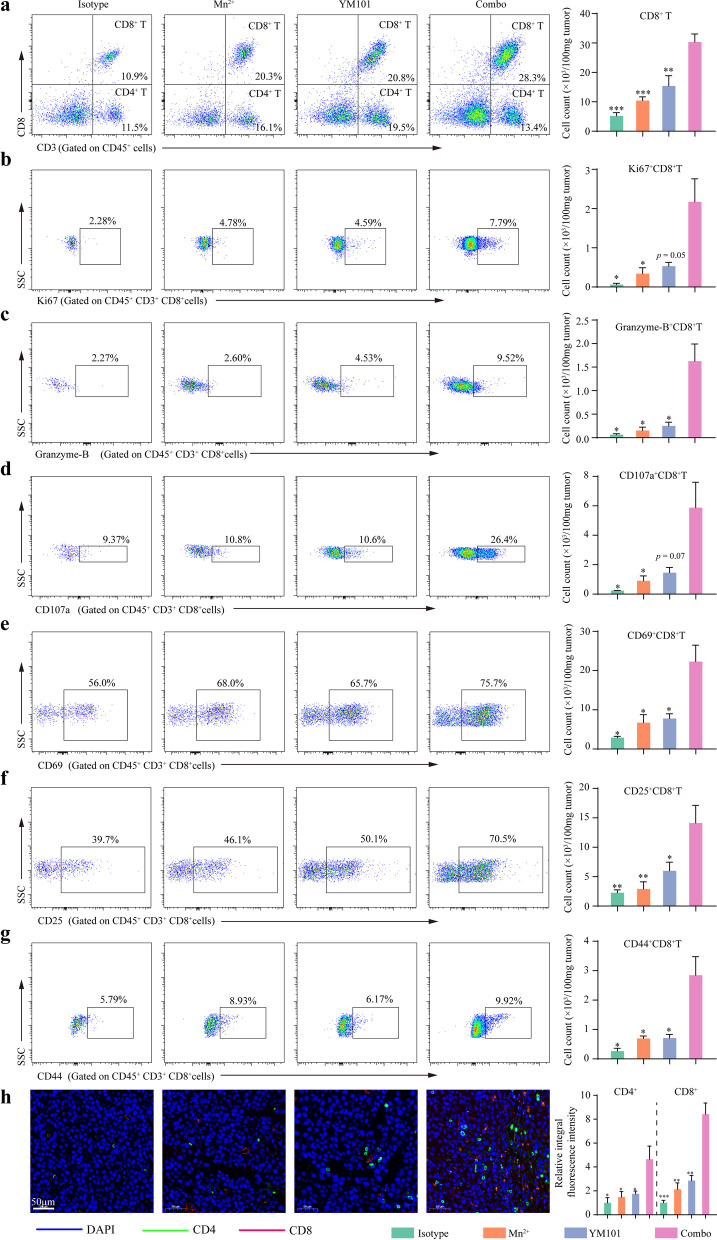
Flow cytometry and immunofluorescent staining assays to analyze the status of CD8^+^ tumor-infiltrating lymphocytes (TILs) in the B16 model. The representative images of tumor-infiltrating **a** CD8^+^ T cells, **b** Ki67^+^ CD8^+^ T cells, **c** Granzyme-B^+^ CD8^+^ T cells, **d** CD107a^+^ CD8^+^ T cells, **e** CD69^+^ CD8^+^ T cells, **f** CD25^+^ CD8^+^ T cells, and **g** CD44^+^ CD8^+^ T cells in flow cytometry assays. **h** The representative images of tumor-infiltrating CD4^+^ and CD8^+^ T cells in immunofluorescent staining assays (n = 5 biological replicates). *p < 0.05, **p < 0.01, ***p < 0.001, and ****p < 0.0001 denote the significant difference relative to combination treatment

Then, in the B16 model, we explored the effect of Mn^2+^ plus YM101 on multiple components in the TME. B16 was a cold or immune-desert tumor model with rare TILs in baseline or even after YM101 treatment. However, Mn^2+^ plus YM101 therapy significantly upregulated the quantity and function of TILs. Flow cytometry results showed that the density of tumor-infiltrating CD8^+^ T cells was increased in the combination therapy group (Fig. 6a). Besides, the densities of Ki67^+^ CD8^+^, Granzyme-B^+^ CD8^+^, and CD107a^+^ CD8^+^ T cells were markedly upregulated in the combination therapy group, indicating the higher tumor-killing capability (Fig. 6b–6d). Moreover, the densities of activated CD8^+^ T cell (CD69^+^ CD8^+^ and CD25^+^ CD8^+^) and memory CD8^+^ T cell (CD44^+^ CD8^+^) were increased in the Mn^2+^ plus YM101 group (Fig. 6e–6g). The following IF assays showed Mn^2+^ plus YM101 therapy promoted immune cell infiltration, converting immune-desert to immune-inflamed phenotype (Fig. 6h).

**Fig. 7:**
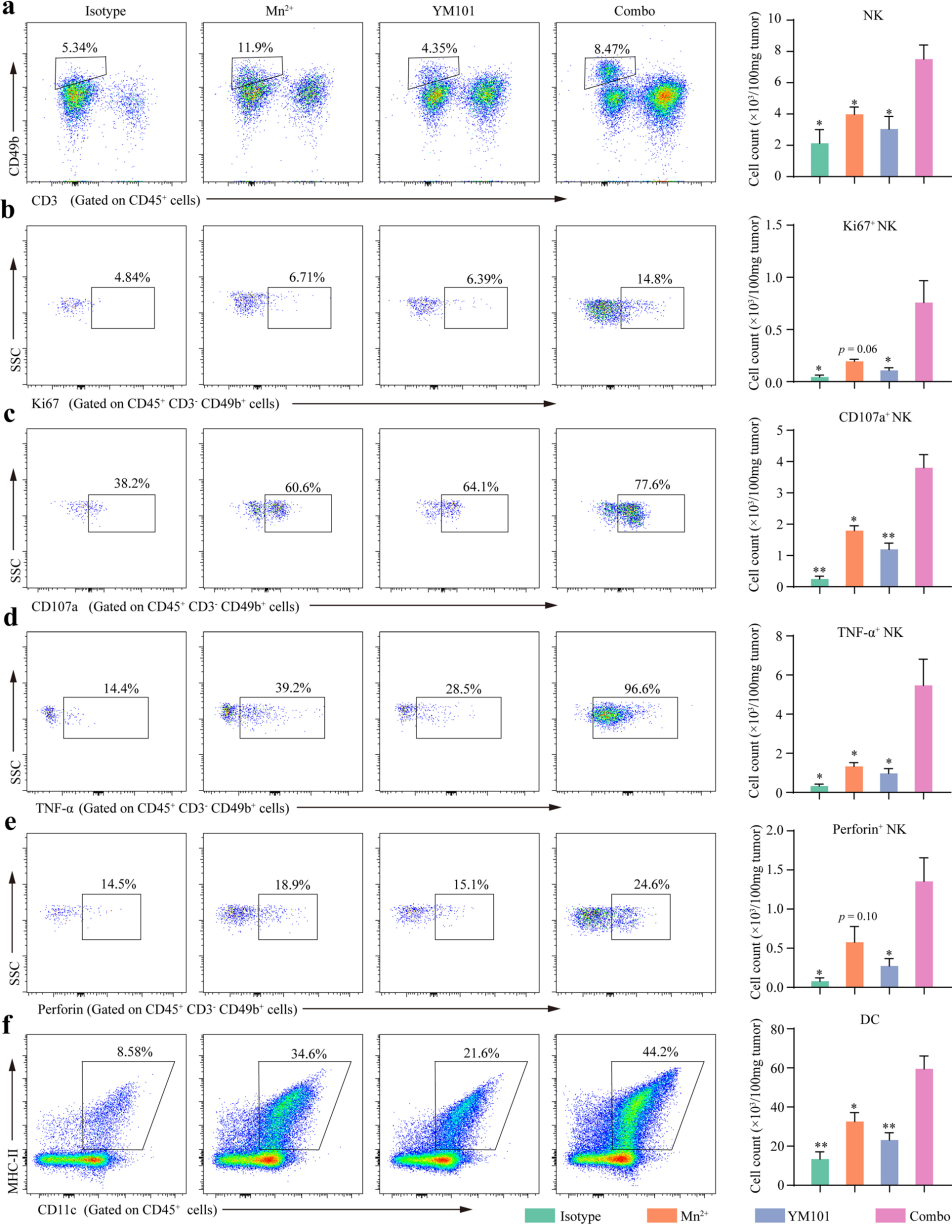
Flow cytometry assays to analyze the status of tumor-infiltrating natural killer cells (NKs) and dendritic cells (DCs) in the B16 model. The representative images of tumor-infiltrating **a** NKs, **b** Ki67^+^ NKs, **c** CD107a^+^ NKs, **d** TNF-α^+^ NKs, **e** Perforin^+^ NKs, and **f** DCs. *p < 0.05, **p < 0.01, ***p < 0.001, and ****p < 0.0001 denote the significant difference relative to combination treatment

Apart from CD8^+^ T cell, NK also participates in the tumor-killing activity. We found that the density of NK (CD3− CD49b+) was highest in Mn^2+^ plus YM101 group (Fig. 7a). Additionally, the densities of Ki67^+^ NK, CD107a^+^ NK, TNF-α^+^ NK, and Perforin^+^ NK were markedly increased in the tumors of mice treated by Mn^2+^ plus YM101 (Fig. 7b-7e). Notably, the Mn^2+^ plus YM101 group had the highest density of DC (CD11c^+^ I-A/I-E^+^) (Fig. 7f). Overall, Mn^2+^ plus YM101 strategy effectively overcame the poor immunogenicity-caused treatment resistance and tuned an immunosupportive microenvironment.

### Mn^2+^ plus YM101 treatment promoted the gene expression related to innate immunity and adaptive immunity

**Fig. 8:**
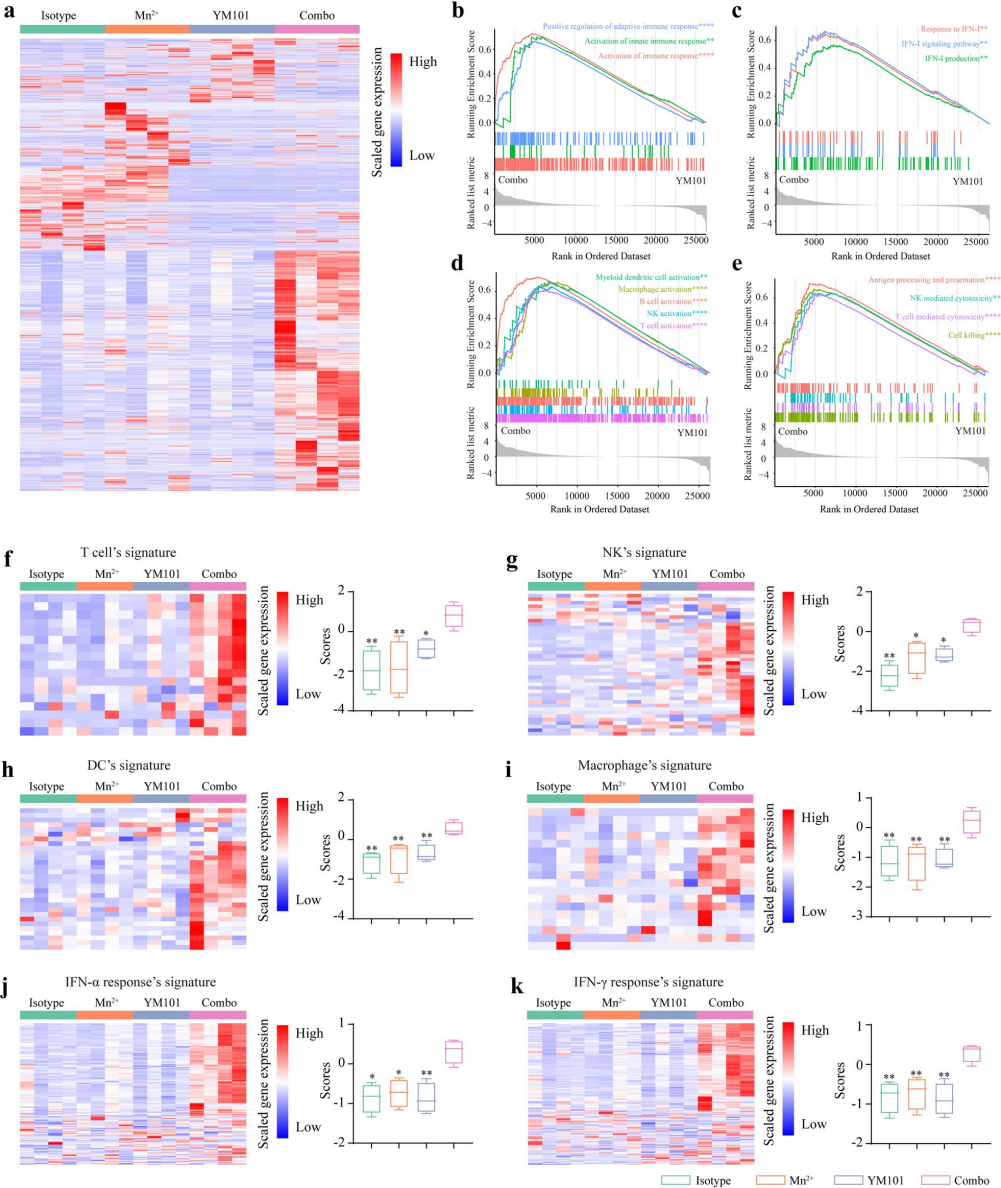
RNA-seq assay to investigate the immune profile of the B16 tumors. **a** The heatmap presenting the relative expression levels of differentially expressed genes (n = 4 biological replicates). **b–e** Gene set enrichment analysis (GSEA) showing the significantly enriched biological processes in Mn^2+^ plus YM101-treated tumors, relative to YM101-treated tumors. The biological processes including activation of immune response, activation of innate immune response, positive regulation of adaptive immune response, IFN-I production, IFN-I signaling pathway, response to IFN-I, T cell activation, NK activation, B cell activation, macrophage activation, myeloid dendritic cell activation, cell killing, T cell mediated cytotoxicity, NK mediated cytotoxicity, and antigen processing and presentation were significantly enriched in the combination group. **f–k** The scores of signatures of T cell, NK, dendritic cell (DC), macrophage, IFN-α response, and IFN-γ response. *p < 0.05, **p < 0.01, ***p < 0.001, and ****p < 0.0001 denote the significant difference relative to combination treatment

To explore the effect of the combination therapy on the expression of immunity-related genes, we performed RNA-seq assays using B16 tumor tissues (Additional File 2). Compared to Isotype, Mn^2+^, and YM101, Mn^2+^ plus YM101 group had a distinct gene expression profile. In general, the expression profile of the Isotype group was similar to that of the Mn^2+^ group, while the YM101 group shared a partially similar gene profile with Mn^2+^ plus YM101 group (Fig. 8a). The results of GSEA showed that the Mn^2+^ plus YM101 group had multiple significantly enriched biological processes, including activation of innate and adaptive immune response, IFN-I production and signaling pathway, activation of multiple immune cells, immune cell-mediated cytotoxicity, as well as antigen processing and presentation, relative to the YM101 group (Fig. 8b–8e).

To comprehensively assess the effect of Mn^2+^ plus YM101 therapy on the TME, we constructed multiple immune signatures [46]. The results showed that the scores of antitumor-associated immune signatures were highest in the Mn^2+^ plus YM101 group (Fig. 8f-8k). The transcriptomic data indicated that Mn^2+^ plus YM101 treatment had a positive effect on innate immunity and adaptive immunity, boosting multiple steps of the antitumor immune response.

### Mn^2+^ activated STING pathway and YM101 reversed epithelial mesenchymal transition (EMT) in vivo

**Fig. 9:**
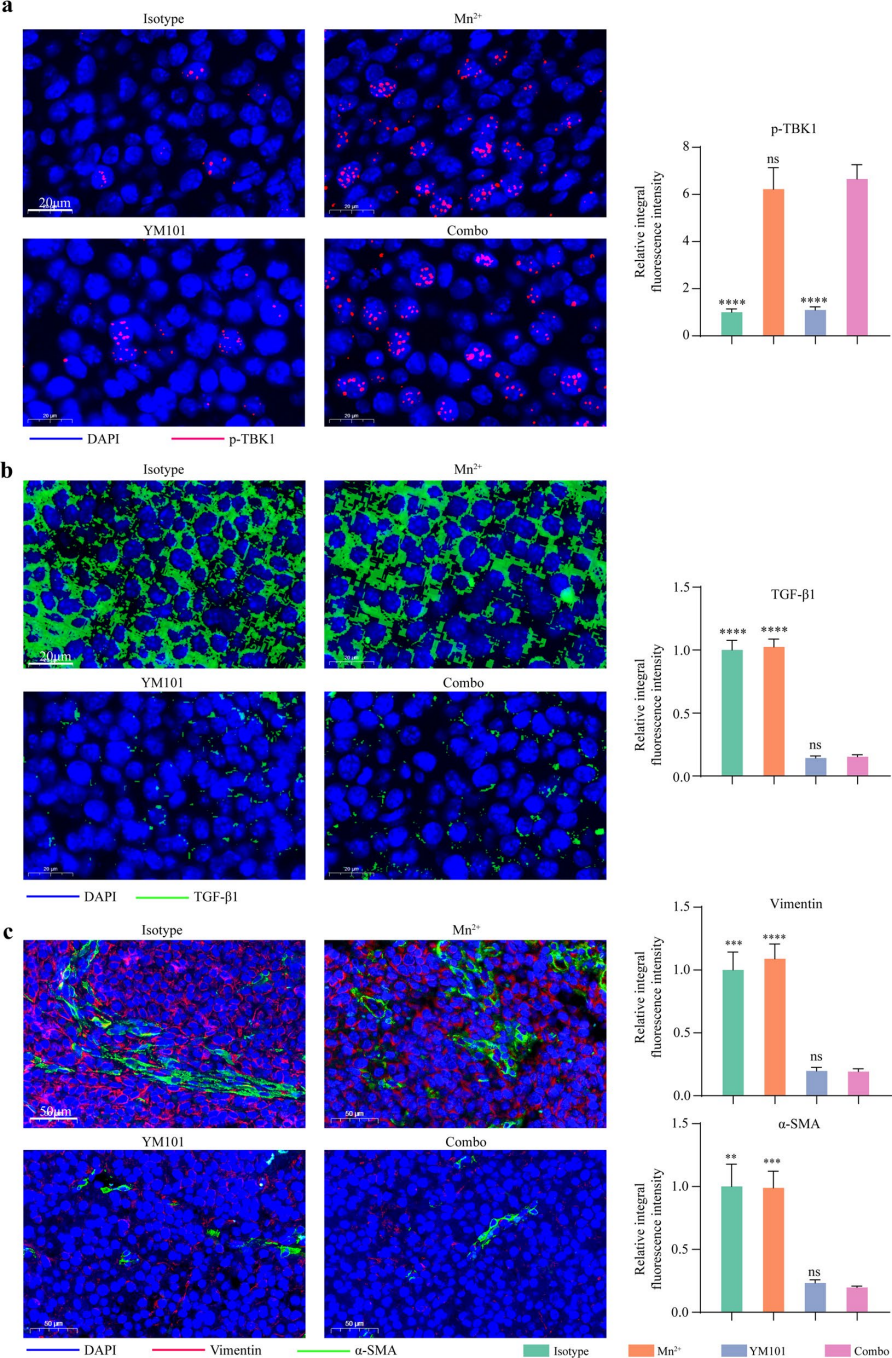
Immunofluorescent staining assays to measure the status of STING pathway, TGF-β signal, and epithelial-mesenchymal transition in the B16 model. The representative images of **a** α-p-TBK1 staining, **b** α-TGF-β1 staining, **(c)** α-SMA and α-Vimentin staining (n = 5 biological replicates). *p < 0.05, **p < 0.01, ***p < 0.001, and ****p < 0.0001 denote the significant difference relative to combination treatment

To validate the effect of Mn^2+^ on STING pathway in vivo, we performed α-p-TBK1 IF assays. The results of IF assays showed that Mn^2+^ treatment activated STING pathway in the B16 model: The level of p-TBK1 was higher in the Mn^2+^ group and the Mn^2+^ plus YM101 group (Fig. 9a). α-SMA is the marker of CAF, and Vimentin is the marker of epithelial-mesenchymal transition. The overexpression of α-SMA and Vimentin is related to active TGF-β signaling, which could be reversed by α-TGF-β moiety. Consistent with our previous work, YM101 and Mn^2+^ plus YM101 treatment effectively cleared the TGF-β1 in the TME and reduced the expression of α-SMA and Vimentin (Fig. 9b and 9c).

## Discussion

According to the TME status, tumors could be divided into three phenotypes: immune-inflamed, immune-desert, and immune-excluded. Among the three phenotypes, immune-inflamed tumors are most likely to respond to α-PD-1/PD-L1 treatment. On the contrary, immune-desert and immune-excluded tumors are non-inflamed tumors, which rarely respond to α-PD-1/PD-L1 therapy. Immune-desert tumors result from immunological ignorance or lacking appropriate T cell priming [5]. Promoting immunogenic cancer cell death and enhancing APC’s function relieve the resistance to α-PD-1/PD-L1 in immune-desert tumors [28]. Immune-excluded tumors are mainly caused by abnormal vascular barriers, increased stromal collagen or fibronectin, and immunosuppressive chemokine panel [5]. Normalizing dysregulated TGF-β signal and angiogenesis in the TME promotes the T cell infiltration into tumors, reverting immune-excluded to immune-inflamed tumors [47–49]. Multi-omics signature study in hepatocellular carcinoma revealed that immune cell function, not immune cell infiltration in the TME, determined immunotherapeutic response [50]. Due to the heterogeneity of the TME, a majority of patients are resistant to α-PD-1/PD-L1 monotherapy [51–53]. For the non-responders, the PD-1/PD-L1 pathway is not the predominant immune defect in the TME, and blocking PD-1/PD-L1 is insufficient to break immune tolerance and trigger immune killing. Hereto, accumulating combination strategies are developed to improve α-PD-1/PD-L1 efficacy and alleviate treatment resistance by enhancing immunogenic cancer cell death, promoting antigen presentation, and rescuing other dysfunctional effectors [54–57].

In the TME, STING pathway plays a vital role in cross-presentation and priming. The development of STING agonists has been a hot issue in cancer therapeutics. DMXAA is the first agent activating murine STING pathway but failed in phase III clinical study [58]. Further investigation showed that the interaction between human STING and DMXAA was weak [59]. Besides, other traditional STING agonists such as cGAMP also have some drawbacks, such as low stability and poor transmembrane capability. Although some novel STING agonists have been developed in the past decade [32, 60], Mn^2+^ has a significant advantage in manufacturing cost and well-studied toxicity. As a natural and easily available STING agonist, Mn^2+^ might be a promising adjuvant drug of immunotherapy.

Manganese is a trace element participating in multiple physiological activities, including reproduction, development, energy metabolism, and antioxidant defenses [61]. Manganese also regulates innate immunity by sensitizing STING pathway [33]. The concentration of manganese in mammalian tissue ranges from 0.3 to 2.9 μg/g wet tissue [62]. According to the recommendations of Dietary Reference Intake, 2 mg/day manganese is an adequate intake for adults, and the tolerable upper intake level is 9–11 mg/day [61]. The gastrointestinal tract negatively regulates the absorption of manganese: High manganese intake decreases intestinal absorption but increases biliary and pancreatic excretion [63]. The manganese turnover is rapid, and the mean retention is 10 days after digestion [64]. It has been reported that 2 μM Mn^2+^ endowed THP1 (human monocyte line) with antivirus capability in vitro [33]. In the present study, we found Mn^2+^ treatment promoted DC maturation and antigen presentation capability. Besides, it was reported 5 mg/kg Mn^2+^ treatment enhanced the efficacy of α-PD-1/PD-L1 in murine tumor models [36]. Additionally, another preclinical study showed that Mn^2+^-based nanoparticles boosted antitumor immunity by activating STING pathway [65]. Notably, the results of a phase I clinical study showed that Mn^2+^ intranasal administration or inhalation augmented the effect of chemoimmunotherapy, even in patients previously failing to chemotherapy combined with α-PD-1 treatment [36]. Importantly, Mn^2+^ treatment had high biosafety, and no manganese overdose-associated toxicity was observed at follow-up [36].

**Fig. 10:**
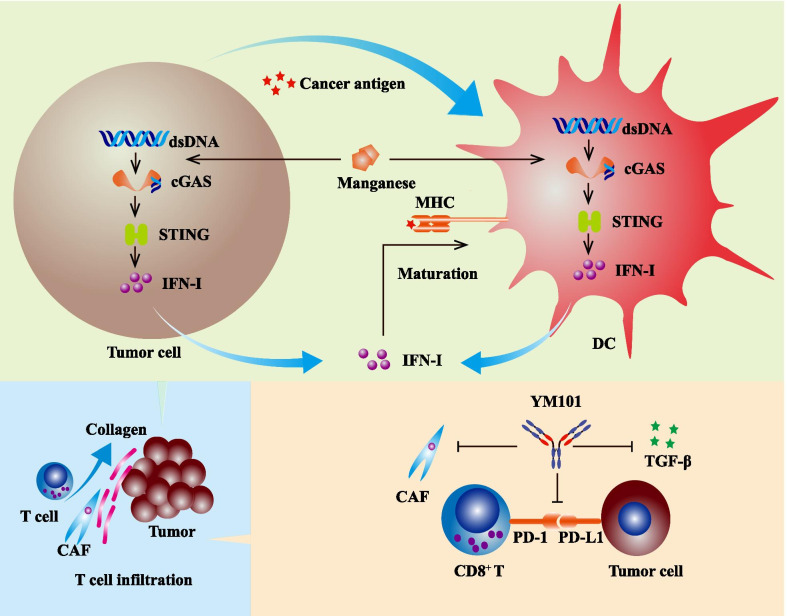
Schematic diagram showing the synergistic effect between Mn^2+^ and YM101. On the one hand, Mn^2+^ promoted dendritic cell (DC) maturation. On the other hand, YM101 restrained cancer-associated fibroblast (CAF) activity, enhanced T cell infiltration and restored T cell from exhaustion. The effects of Mn^2+^ and YM101 were independent and complementary, and the combination therapy had a potent and broad-spectrum antitumor effect

In this study, we investigated the efficacy of Mn^2+^ plus α-TGF-β/PD-L1 BsAb YM101, which simultaneously activated STING pathway and suppressed TGF-β and PD-1/PD-L1 signals (Fig. 10). This novel cocktail strategy had a broader antitumor spectrum and more potent antitumor activity than YM101 monotherapy. Specifically, although YM101 effectively retarded tumor growth in some immune-excluded tumor models, its antitumor effect was limited in the immune-desert tumor model. The combination therapy dramatically boosted the efficacy of YM101, especially in the poorly immunogenic tumor. The flow cytometry and RNA-seq data showed that the combination treatment significantly upregulated the densities and functions of tumor-infiltrating CD8^+^ T and NK cells. Besides, a series of innate and adaptive immunity-related biological processes were markedly enriched after the combination therapy. Our results indicated that this combination strategy successfully overcame the weak immunogenicity-caused treatment resistance and effectively reinvigorated adaptive antitumor immunity by stimulating innate immunity.

It was reported that Mn^2+^ plus α-PD-1/PD-L1 had better antitumor efficacy than α-PD-1/PD-L1 monotherapy [36]. In the present study, we confirmed this synergistic effect. However, in immune-excluded tumor model, the antitumor effect of Mn^2+^ plus α-PD-1/PD-L1 was modest, significantly weaker than that of Mn^2+^ plus YM101. Our previous work showed that in EMT-6 model, hyperactive TGF-β signaling in the TME was related to increased collagen deposition and hampered T cell infiltration [27]. Blocking TGF-β promoted T cell infiltration and improved the effect of α-PD-1/PD-L1 [27]. Consistently, Mn^2+^ plus YM101 promoted T cell penetration into tumors. This shift from immune-excluded to immune-inflamed might contribute to the enhanced antitumor effect of Mn^2+^ plus YM101 therapy in high TGF-β models.

The cocktail strategy of Mn^2+^ plus YM101 is a potent and broad-spectrum antitumor immunotherapy. In the real world, combination therapy means a higher risk of immune-related adverse events. Although we had not observed treatment-associated toxicity in murine tumor models, further explorations are needed to confirm the safety and efficacy of this combination therapy.

## Conclusion

In this study, we developed a novel combination therapy of Mn^2+^ and YM101, which simultaneously boosted innate and adaptive immunity. This novel cocktail strategy had a superior antitumor effect to YM101 monotherapy and Mn^2+^ plus α-PD-1/PD-L1 therapy. Further investigation indicated that Mn^2+^ plus YM101 treatment promoted the transformation from non-inflamed to immune-inflamed tumor: restraining CAF activity and collagen production, increasing the infiltration of effector cells, enhancing the tumor-killing activities of T cells and NKs, strengthening the antigen presentation of APCs, and upregulating the ratio of memory T cells. Generally, this combination therapy exhibited a broad antitumor spectrum even in immune-excluded and immune-desert tumors. We believed this Mn^2+^-based combination therapy could greatly relieve α-PD-1/PD-L1 resistance and become a universal immunotherapy strategy across all three phenotypes of tumors.

## Supplementary Information

**Additional file 1.** Supplementary table and figures.

**Additional file 2.** RNA-seq profile.

## Data Availability

The dataset generated during the current study is available from the corresponding author on reasonable request.

## Abbreviations

BMDC: Bone marrow-derived dendritic cell
BsAb: Bispecific antibody
CAF: Carcinoma-associated fibroblast
CFSE: Carboxyfluorescein diacetate succinimidyl ester
DC: Dendritic cells
DEG: Differentially expressed gene
EMT: Epithelial-mesenchymal transition
ELISA: Enzyme-linked immunosorbent assay
GSEA: Gene set enrichment analysis
IFN: Interferon
MHC: Major histocompatibility complex
MLR: Mixed lymphocyte reaction
MoDC: Monocyte-derived dendritic cell
PBMC: Peripheral blood mononuclear cell
PD-1: Programmed cell death protein 1
TGF-β: Transforming growth factor-beta
TIL: Tumor-infiltrating lymphocytes
TME: Tumor microenvironment
Treg: Regulatory T
STING: Stimulator of interferon genes
