# Supplementary material for: Combine and conquer: manganese synergizing anti-TGF-β/PD-L1 bispecific antibody YM101 to overcome immunotherapy resistance in non-inflamed cancers
Source: J Hematol Oncol. 2021 Sep 15;14:146. doi: 10.1186/s13045-021-01155-6 (PMC8442312; doi:10.1186/s13045-021-01155-6)
Supplement: Supplementary file 1 — Additional file 1. Supplementary table and figures. [file 13045_2021_1155_MOESM1_ESM.docx]

**Combine and conquer: manganese synergizing anti-TGF-β/PD-L1 bispecific antibody YM101 to overcome immunotherapy resistance in non-inflamed cancers**

Ming Yi^1^, Mengke Niu^1^, Jing Zhang^2^, Shiyu Li^1^, Shuangli Zhu^1^, Yongxiang Yan^2^, Ning Li^3^, Pengfei Zhou^2^, Qian Chu^1^, Kongming Wu^1,3*^

1. Department of Oncology, Tongji Hospital of Tongji Medical College, Huazhong University of Science and Technology, 1095 Jiefang Avenue, Wuhan, 430030, People's Republic of China.
2. Wuhan YZY Biopharma Co., Ltd, Biolake, C2-1, No.666 Gaoxin Road, Wuhan, 430075, People's Republic of China.
3. Department of Medical Oncology, The Affiliated Cancer Hospital of Zhengzhou University & Henan Cancer Hospital, Zhengzhou, 450008, People's Republic of China.

*** Corresponding authors**

Kongming Wu, Department of Oncology, Tongji Hospital of Tongji Medical College, Huazhong University of Science and Technology, 1095 Jiefang Avenue, Wuhan, 430030, People's Republic of China. E-mail: wukm_lab@163.com.

**Author E-mails:**

Ming Yi: d201981836@hust.edu.cn;

Mengke Niu: niumengke9505@163.com;

Jing Zhang: zhangjing@yzybio.com;

Shiyu Li: li_shiyu17@qq.com;

Shuangli Zhu: 1667985477@qq.com;

Yongxiang Yan: yanyongxiang@yzybio.com;

Ning Li: lining97@126.com

Pengfei Zhou: pfzhou@yzybio.com;

Qian Chu: qianchu@tjh.tjmu.edu.cn;

Kongming Wu: wukm_lab@163.com.

**Figure Legends**

**Figure S1: Mn^2+^ treatment synergized YM101 in one-way mixed lymphocyte reaction assay.** In MLR assays, BMDCs from BALB/c mice were used as responder cells, and splenocytes from C57BL/6 mice were used as responder cells. The ratio of responder to stimulator was 2:1. Then, TGF-β1 and antibodies were added to the mixed cells. After culture for 96 hours, the ratio of daughter CD4^+^ T cell was measured by CFSE dilution assays. **p* < 0.05, ***p* < 0.01, ****p* < 0.001, and *****p* < 0.0001 denote the significant difference relative to the last group when not marked with lines.


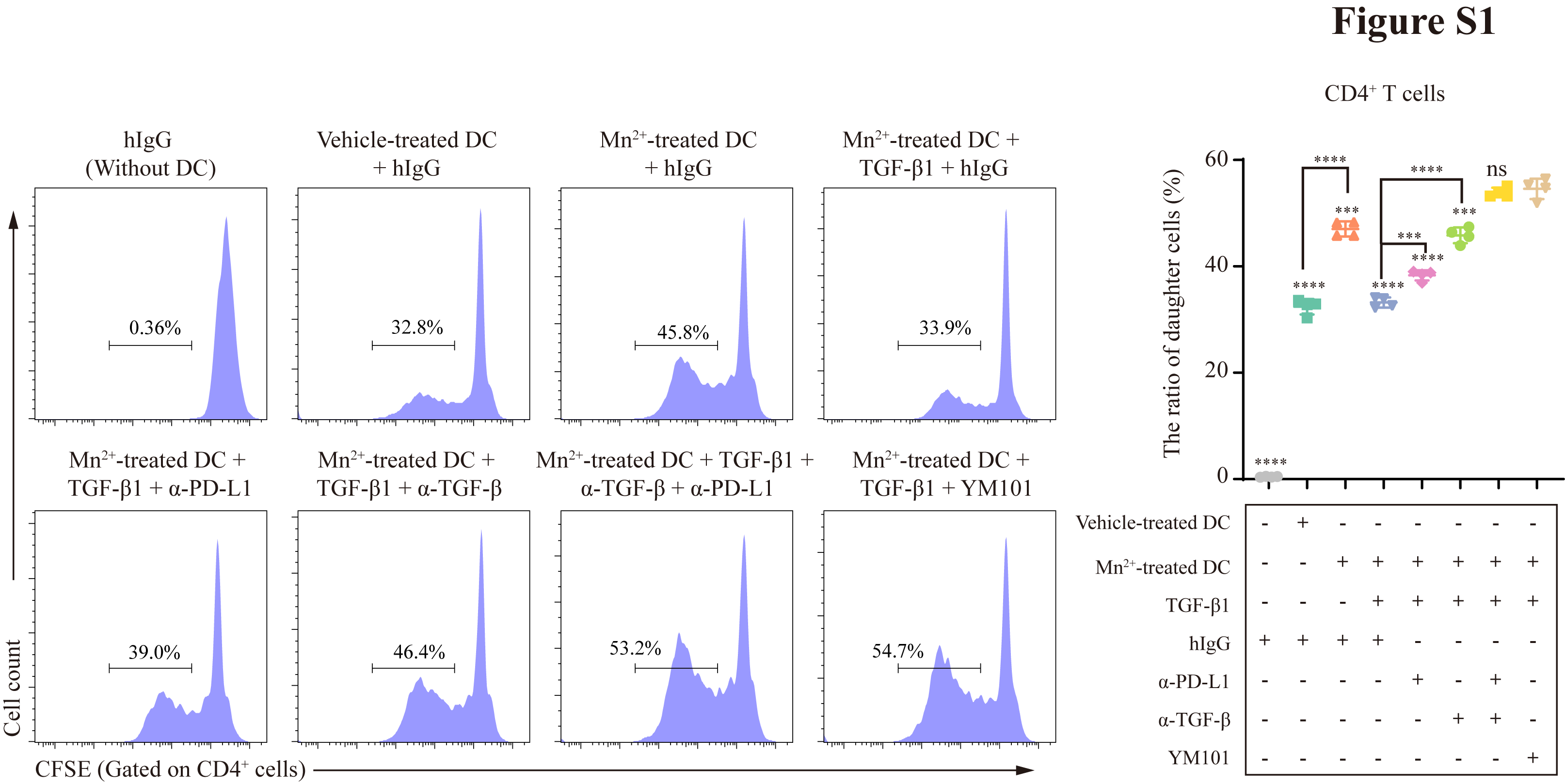


**Figure S2:** **Manganese plus YM101 therapy decreased peritumoral collagen production.** The representative images of α-collagen I immunofluorescence staining in the tumor periphery.


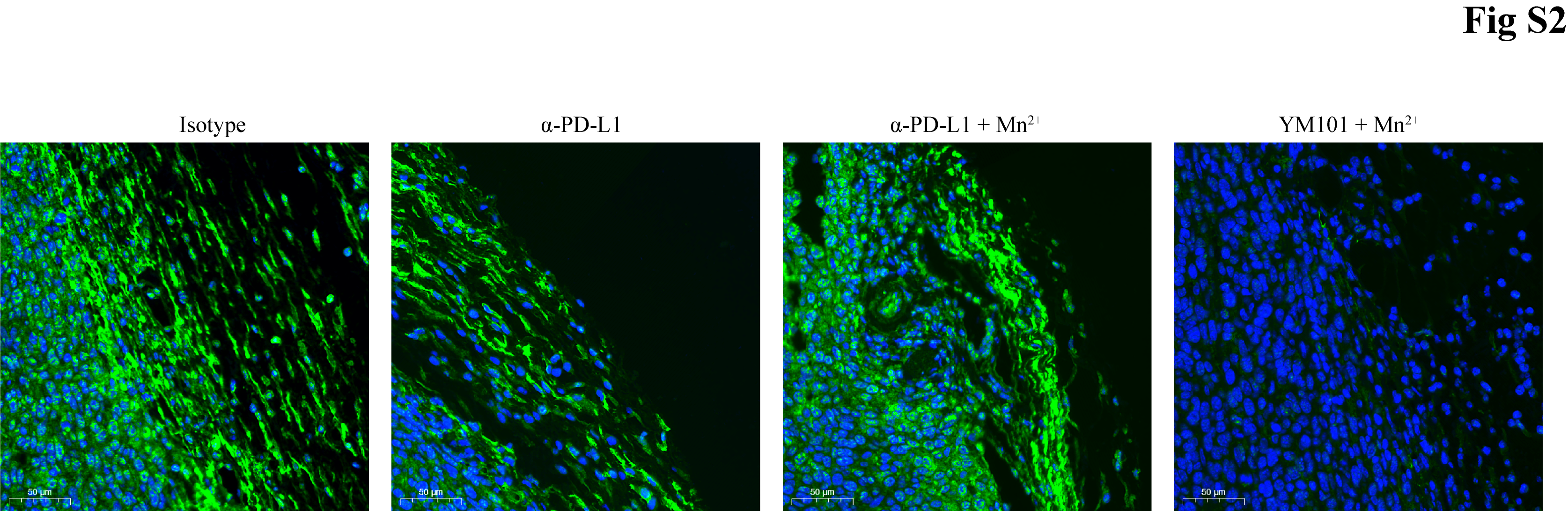


**Table S1: Gene lists of signatures in RNA-seq assay.** The immune signatures were constructed referring to previous reports [1, 2].

| Immune signatures | Genes |
| --- | --- |
| T cell | *Bcl11b, Cd2, Cd28, Cd3d, Cd3e, Cd3g, Cd6, Cd96, Gimap3, Gimap5, Itm2a, Lck, Ncald, Prkcq, Sh2d1a, Skap1, Trat1* |
| NK | *Bzrap1, Cacna2d2, Cd247, Cd7, Colq, Ctsw, Dll1, Eomes, Fam179a, Fasl, Fez1, Gk5, Gpr56, Gtf3c1, Gzmd, Gzme, Gzmf, Gzmg, Gzmm, Il12rb2, Il2rb, Kir3dl1, Kir3dl2, Klrc1, Klrc2, Klrc3, Klrd1, Lair1, Myom2, Ncald, Nkg7, Phldb2, Plekhf1, Prf1, Samd3, Stat4, Tbc1d31, Tbx21, Tigit, Tox, Tsen54, Txk, Yes1* |
| Dendritic cell | *C1qc, Ccdc88a, Ccl2, Ccl3, Cd300e, Cd86, Clec1a, Clec4b1, Clec5a, Csf2ra, Fam49a, Fcgr1, Fn1, Fstl1, Gpx3, H2-Aa, Inhba, Lgmn, Ngfr, Pdgfrl, Pdpn, Prkar2b, Siglec1, Slamf9, Stab1, Thbd, Tnfaip2, Ttyh2, Ubd, Vcam1* |
| Macrophage | *Aif1, Ccl1, Ccl26, Cd163, Cd300lb, Cnr1, Cnr2, Cpm, Csf3r, Eng, Igf1, Il34, L1cam, Lilra5, Lrp1, Ms4a7, Trem1, Treml1* |
| IFN-α response | *1700057G04Rik, 2200002J24Rik, AC132444.1, AC168977.1, Adar, B2m, Batf2, Bst2, C1s, Casp1, Casp8, Ccrl2, Cd47, Cd74, Cmpk2, Cmtr1, Cnp, Csf1, Cxcl10, Cxcl11, Cxcl15, Ddx60, Dhx58, Eif2ak2, Elf1, Epsti1, Fam46a, Fibin, Gbp11, Gbp2, Gbp3, Gbp4, Gbp5, Gbp7, Gbp8, Gbp9, Gm11127, Gm7030, Gm8909, Gmpr, Gmpr2, H2-D1, H2-K1, H2-M1, H2-M10.1, H2-M10.2, H2-M10.3, H2-M10.4, H2-M10.5, H2-M10.6, H2-M11, H2-M2, H2-M3, H2-M5, H2-M9, H2-Q1, H2-Q10, H2-Q2, H2-Q4, H2-Q6, H2-Q7, H2-T10, H2-T22, H2-T23, H2-T24, H2-T3, Helz2, Herc3, Herc6, I830012O16Rik, Ifi27, Ifi27l2a, Ifi27l2b, Ifi35, Ifi44, Ifih1, Ifit2, Ifit3, Ifitm1, Ifitm2, Ifitm3, Ifitm5, Ifitm6, Ifitm7, Il15, Il4ra, Il7, Irf1, Irf2, Irf7, Irf9, Isg15, Isg20, Lamp3, Lap3, Lgals3bp, Lpar6, Ly6e, Mov10, Mvb12a, Mx2, Ncoa7, Nmi, Nub1, Oas1a, Oas1b, Oas1c, Oas1d, Oas1e, Oas1f, Oas1g, Oas1h, Oas2, Oas3, Oasl1, Oasl2, Ogfr, Parp12, Parp14, Parp9, Plscr1, Plscr2, Plscr5, Pnpt1, Procr, Psma3, Psmb11, Psmb5, Psmb8, Psmb9, Psme1, Psme2, Psme2b, Ripk2, Rnf31, Rsad2, Rtp4, Samd9l, Sell, Slc25a28, Sp110, Stat2, Tap1, Tdrd7, Tmem140, Trafd1, Trim12a, Trim12c, Trim14, Trim21, Trim25, Trim26, Trim30a, Trim30b, Trim30d, Trim31, Trim34a, Trim34b, Trim43a, Trim43b, Trim43c, Trim5, Trim6, Txnip, Uba1, Uba1y, Uba7, Ube2l6, Usp18, Wars* |
| IFN-γ response | *1700057G04Rik, 2010002M12Rik, 2200002J24Rik, 4922502D21Rik, 4930525M21Rik, AC132444.1, AC168977.1, Adar, Apol6, Apol9a, Arid5b, Arl4a, Arl4c, Arl4d, B2m, Bank1, Batf2, Bpgm, Bst2, Btg1, C1ra, C1rb, C1s, Casp1, Casp12, Casp3, Casp4, Casp7, Casp8, Ccl11, Ccl12, Ccl2, Ccl5, Ccl7, Ccl8, Cd274, Cd38, Cd40, Cd69, Cd74, Cd86, Cdkn1a, Cfb, Cfh, Cfhr1, Cfhr2, Ciita, Cmklr1, Cmpk2, Cmtr1, Csf2rb, Csf2rb2, Cxcl10, Cxcl11, Cxcl15, Cxcl9, Ddx58, Ddx60, Dhx58, Eif2ak2, Eif4e3, Epas1, Epsti1, Fas, Fbxo39, Fcgr1, Fgl2, Fibin, Fpr-rs3, Fpr-rs4, Fpr-rs6, Fpr1, Fpr2, Fpr3, Gbp11, Gbp2, Gbp3, Gbp4, Gbp5, Gbp7, Gbp8, Gbp9, Gch1, Gm11127, Gm14446, Gm4788, Gm7030, Gm8909, Gpr18, Gzma, H2-Aa, H2-Ab1, H2-D1, H2-DMa, H2-Eb1, H2-Eb2, H2-K1, H2-M1, H2-M10.1, H2-M10.2, H2-M10.3, H2-M10.4, H2-M10.5, H2-M10.6, H2-M11, H2-M2, H2-M3, H2-M5, H2-M9, H2-Q1, H2-Q10, H2-Q2, H2-Q4, H2-Q6, H2-Q7, H2-T10, H2-T22, H2-T23, H2-T24, H2-T3, Helz2, Herc3, Herc6, Hif1a, I830012O16Rik, Icam1, Ido1, Ifi27, Ifi27l2a, Ifi27l2b, Ifi35, Ifi44, Ifih1, Ifit1, Ifit2, Ifit3, Ifitm1, Ifitm2, Ifitm3, Ifitm5, Ifitm6, Ifitm7, Ifnar2, Il10ra, Il15, Il15ra, Il18bp, Il2rb, Il4ra, Il6, Il7, Irf1, Irf2, Irf4, Irf5, Irf7, Irf8, Irf9, Isg15, Isg20, Isoc1, Itgb1, Itgb2, Itgb7, Jak1, Jak2, Klrk1, Lap3, Lats1, Lats2, Lcp2, Lgals3bp, Ly6e, Lysmd2, March1, March8, Mettl7b, Mt1, Mt2, Mthfd2, Mthfd2l, Mvp, Mx2, Myd88, Nampt, Ncoa2, Ncoa3, Nfkb1, Nfkb2, Nfkbia, Nlrc5, Nmi, Nod1, Nup93, Oas1a, Oas1c, Oas1f, Oas1g, Oas1h, Oas2, Oas3, Oasl1, Oasl2, Ogfr, P2ry14, Parp12, Parp14, Pde4a, Pde4b, Pde4c, Pde4d, Peli1, Peli2, Pfkl, Pfkm, Pfkp, Pim1, Pim3, Pla2g4a, Plscr1, Plscr2, Plscr5, Pml, Pnp, Pnp2, Pnpt1, Psma2, Psma3, Psmb10, Psmb11, Psmb2, Psmb5, Psmb8, Psmb9, Psme1, Psme2, Psme2b, Ptgs1, Ptgs2, Ptpn1, Ptpn11, Ptpn2, Ptpn6, Rapgef6, Rbck1, Ripk1, Ripk2, Rnf213, Rnf31, RP23-322E15.2, Rsad2, Rtp4, Samd9l, Samhd1, Sectm1a, Sectm1b, Selp, Serping1, Slamf7, Slc25a28, Socs1, Socs3, Sod2, Sp110, Sppl2a, Sri, Sspn, St3gal5, St8sia4, Stat1, Stat2, Stat3, Stat4, Tap1, Tapbp, Tdrd7, Tnfaip2, Tnfaip3, Tnfaip6, Tnfsf10, Tor1a, Tor1b, Trafd1, Trim14, Trim21, Trim25, Trim26, Trim31, Trim43a, Trim43b, Trim43c, Txnip, Tyk2, Ube2l6, Upp1, Upp2, Usp18, Vamp5, Vamp8, Vcam1, Wars, Xaf1, Xcl1, Zbp1, Znfx1* |

**Reference**

1. Yi M, Zhang J, Li A, Niu M, Yan Y, Jiao Y, et al. The construction, expression, and enhanced anti-tumor activity of YM101: a bispecific antibody simultaneously targeting TGF-β and PD-L1. J Hematol Oncol. 2021;14:27.

2. Lan Y, Zhang D, Xu C, Hance KW, Marelli B, Qi J, et al. Enhanced preclinical antitumor activity of M7824, a bifunctional fusion protein simultaneously targeting PD-L1 and TGF-β. Sci Transl Med. 2018;10.
